# Quality evaluation of *Panax quinquefolium* from different cultivation regions based on their ginsenoside content and radioprotective effects on irradiated mice

**DOI:** 10.1038/s41598-018-37959-9

**Published:** 2019-01-31

**Authors:** Dengqun Liao, Chan Jia, Peng Sun, Jianjun Qi, Xian’en Li

**Affiliations:** Institute of Medicinal Plant Development, Chinese Academy of Medical Sciences & Peking Union Medical College, Beijing, 100193 PR China

## Abstract

Ginsenosides are one of major types of bioactive compounds in American ginseng (AG) and utilized to assess the quality of various AG samples. The contents of ginsenosides showed cultivation region-related variation, which is possibly associated with AG’s pharmacological effect difference. Therefore, to reveal the quality difference of AGs in different cultivation regions, AG samples from seven cultivation regions were evaluated via analyzing their contents of nine ginsenosides and the biochemical parameters in AG-treated irradiated mice. Pre-administration of AG decoctions could reversely modulate the irradiation-induced changes of antioxidant enzymatic activity, cytokine level and hormone level in irradiated mice, which demonstrated that AG had the radioprotective effects due to its antioxidative, immunomodulatory and anti-inflammatory properties. However, this radioprotection effect varied among different cultivation regions of AGs. Collectively, Beijing and Canada-cultivated AGs had the best radioprotection. Heilongjiang and Jilin-originated AGs had the similar pharmacological effects while USA, Shandong and Shaanxi-grown AGs had closer pharmacological effects. This biochemical measurements-based PCA and heatmap clustering of AGs from seven cultivation regions was nearly consistent with ginsencoside content- and the previous serum metabolome-based analyses. However, the pearson correlation analysis revealed that only Rb3 and Rd were significantly correlated with some of assayed biochemical parameters in irradiated mice pretreated with different cultivation regions of AG extracts.

## Introduction

While ionizing radiation (IR) is increasingly used in the successful diagnosis of many human health problems and alone or combinational therapy of human cancers such as breast cancer^1^, extraabdominal desmoid tumors^2^, lung cancer^3^ and prostate cancer^4^, the public still need to pay the special attentions to the safety and side effects of the intended radiation exposure e.g during the radiotherapy and chest x-ray diagnosis or unwanted radiation exposure, e.g. Fukushima Nuclear Leak. Although the localised irradiation is usually adopted to reduce radiation risk in the radiotherapy, ionizing radiation had adverse effects on neighbor or even distant unirradiated cells due to its bystander and abscopal effects^5^. Meantime, ionizing radiation caused injuries to almost all the organs/tissues in the living organism such as spleen, liver, skin, brain and the gonads, although the probability and severity of ionizing radiation-induced risk often depends on various factors such as radiation dose and time, the health condition and age of the exposed person. To the current radiobiological knowledge, the radiation-induced injuries at the cellular or tissue level are mainly attributed to the oxidative damage on macromolecules DNA, lipids and proteins via the generation of free radicals and reactive oxygen species (ROS)^6^. Concurrently, immune and inflammation responses are also induced by irradiation to be against or adaptive to IR-induced oxidative stress^7^. Therefore, compounds or natural herbal products with antioxidative, immunomodulatory and (or) anti-inflammatory properties were investigated and appraised for their radioprotective performance in counteraction or alleviation of IR-induced side effects^8–10^.

Many preclinical and clinical studies revealed that AG extract and its active compounds such as ginsenosides and polysaccharides possessed the antioxidative, immunomodulatory and anti-inflammatory properties^11–14^. Like other two precious *Panax* species, American ginseng (*Panax quinquefolius* L., Xi yangshen in Chinese) is widely used not only in folk supplementary diet as tonics and food additives but also in clinic to treat cancers, various fatigues, cardiovascular and metabolic diseases and so on. In addition, American ginseng was also prescribed in clinic by some Chinese doctors to improve irradiation-induced active syndromes such as oral mucosa inflammation and ulcer during or post radiotherapy of cancers^15–17^. *Ex-vivo* experiments showed that American ginseng extract could reduce irradiation-induced oxidative stress and DNA damage^18–20^, indicating that it had the radio-protective effects.

American ginseng is now cultivated mainly in its native countries American and Canada and in north provinces of China. A number of chemometric studies revealed that AGs from different cultivation regions had the distinct metabolic compositions and profiles, for example, ginsenosides^21–31^, which are one of its major active compounds. A couple of experimental evidences indicated that AGs with the different metabolic profiles had somehow different pharmacological effects, which was possibly related to ginsenoside variation^32–34^. Studies from *Panax ginseng* Meyer and *Panax notoginseng* demonstrated that their extracts and ginsenosides (Rc, Rd and Rg1 etc.) showed radioprotective effects via attenuation of radiation-induced DNA damage, oxidative stress, and inflammation etc.^35–39^. *Panax ginseng* extract (PGE) could markedly prevent the increases of irradiation-induced hepatic proinflammatory cytokines IL-6 and TNF-α^40^. Therefore, in this paper, we first investigated the variation of nine ginsenosides among AG samples from seven cultivation places: American, Canada and five provinces of China including Heilongjiang, Jilin, Shandong, Shaanxi and Beijing. Then, we investigated and evaluated the radioprotective effects of AGs from these cultivation regions via assays of antioxidative, immune function and hormone parameters in various AG-treated irradiated mice. Finally, the correlation of ginsenoside variation among different cultivation regions of AGs and the assayed individual radioprotective parameters was analyzed to evaluate the potential roles of the varying ginsenoside contents of AGs from different cultivation regions contributing to the radioprotection difference among them.

## Results and Discussion

### Ginsenoside contents in AG roots from different cultivation regions

Ginsenosides are one of major bioactive compounds in American ginseng. Among the isolated ginsenosides, Rb1, Rb2, Rb3, Rc, Rg1, Re and Rd accounted for the majority of the total saponin content in AG roots^41^. These ginsenosides were highly related to AG’s antioxidant, neuroprotective, cardioprotective, antidiabetic and anticancer properties^11,13^. These ginsenosides or most of them were simultaneously quantified by researchers to study the influence of cultivation region and year, extraction and processing on AG quality or ginsenoside content or to distinguish panax species, cultivated and wild AGs^21–31,42,43^. Rb1, Re and Rg1 were abundant in AG roots. The ratios of Rb1: Rg1 (>5), Rg1: Re (<1.0) and protopanaxadiol (PPD)-type to protopanaxatriol (PPT)-type ginsenosides (>2) were used to distinguish American ginseng from Asian ginseng (*Panax ginseng* C. A. Meyer)^13^. Therefore, the contents of these representative ginsenosides in AG samples from seven cultivation regions were determined using the optimized UPLC-UV method (Supplementary Table 1). Rg1, Re, Rg2, Rb1, Rc, Rb2, Rb3, Rd and Rg3 were separately eluted at 6.788 min, 7.014 min, 16.487 min, 17.067 min, 17.839 min, 19.117 min, 19.455 min, 20.273 min and 22.890 min (Supplementary Fig. 1). The measurements (Table 1) showed that Rb1 and Re were the most abundant ginsenosides among nine assayed analytes in AG samples of all seven cultivation regions, which was consistent with the investigations^24,31,32,43,44^. Although some cultivated AGs contained higher Rg1 than Re, which was cultivation region or population-dependent^21,42,44^, Rg1 content in our analyzed samples was about 5–10 times lower than Re and close to Rb2, Rc and Rd. The total content of Rb1, Re and Rg1 ranged from 24.14 ± 0.63 mg/g (SX) to 46.73 ± 7.35 mg/g (Canada), which indicated that AGs that we collected from seven cultivation regions all met the least percentage quality requirement of AGs as qualified medicine (2%)^45^. The contents of Rg2 and Rg3 in AG roots are less studied compared to the other seven ginsenosides. As the reported content of Rg2 and Rg3 in AG roots from Canada or Jilin province of China^24,44,46,47^, much less Rg2 and Rg3 were present in roots of our AG samples.

**Table 1:** Contents of nine ginsenosides in AG roots from seven cultivation regions.

Origin	Rg1 (mg/g)	Rg2 (mg/g)	Rg3 (mg/g)	Rb1 (mg/g)	Rb2 (mg/g)	Rb3 (mg/g)	Rc (mg/g)	Rd (mg/g)	Re (mg/g)	Rg1 + Rb1 + Re (mg/g)
US	1.77 ± 0.48ab	0.13 ± 0.02ab	0.13 ± 0.06	17.69 ± 5.04ab	1.71 ± 0.44	0.36 ± 0.10	1.80 ± 0.49c	2.10 ± 1.09ab	14.60 ± 2.55b	34.06 ± 6.75ab
CA	2.29 ± 0.87b	0.10 ± 0.03a	0.09 ± 0.04	25.92 ± 6.47c	1.56 ± 0.33	0.33 ± 0.08	1.62 ± 0.41bc	2.21 ± 0.75ab	18.52 ± 1.40c	46.73 ± 7.35c
HLJ	1.90 ± 0.68ab	0.15 ± 0.04bc	0.11 ± 0.11	19.31 ± 5.99bc	2.00 ± 0.57	0.29 ± 0.07	1.05 ± 0.19a	1.91 ± 0.44ab	13.00 ± 2.13ab	34.20 ± 8.18ab
JL	1.72 ± 0.38ab	0.18 ± 0.05c	0.05 ± 0.01	18.15 ± 4.38ab	2.18 ± 1.13	0.35 ± 0.08	1.15 ± 0.23ab	2.40 ± 0.69b	15.22 ± 2.80b	35.08 ± 6.69b
SD	1.17 ± 0.37a	0.17 ± 0.01bc	0.07 ± 0.02	17.54 ± 3.69ab	1.41 ± 0.24	0.27 ± 0.06	1.36 ± 0.27abc	1.22 ± 0.41a	12.31 ± 0.92ab	31.03 ± 4.53ab
SX	1.87 ± 0.52ab	0.08 ± 0.01a	0.08 ± 0.02	11.09 ± 1.03a	1.27 ± 0.07	0.27 ± 0.03	1.43 ± 0.13abc	1.05 ± 0.25a	11.18 ± 0.67a	24.14 ± 0.63a
BJ	1.41 ± 0.34ab	0.19 ± 0.00c	0.06 ± 0.00	17.75 ± 0.81ab	1.53 ± 0.18	0.31 ± 0.05	1.55 ± 0.20abc	1.12 ± 0.04a	14.54 ± 0.15b	33.69 ± 1.01ab

Note: Ginsenoside content was expressed as mean ± SD. The data marked with different letters (a, b, c) indicated their significant difference (*P* < 0.05) in AG roots between two compared cultivation regions.

**Figure 1:**
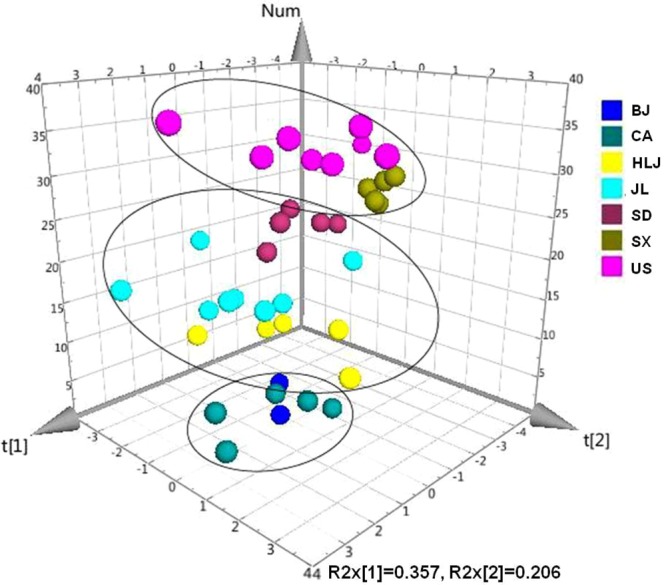
PCA result of 38 AG samples from seven cultivation regions. The first two components revealed 56.3% of the total variance.

Among nine analytes, the contents of Rg3, Rb2 and Rb3 showed no variation among seven cultivation regions. Our results revealed that the concentrations of the remaining six quantified ginsenosides differed just between some of seven cultivation regions. For example, Rg1 was significantly lower only in AGs from Shandong province, China than from Canada. Rc content showed no significant difference among the domestic AGs; its content was only found significantly different between Heilongjiang province (1.05 ± 0.19 mg/g) and the originating countries Canada (1.62 ± 0.41 mg/g) and USA (1.80 ± 0.49 mg/g). In addition, AGs from Shaanxi province of China had the lowest differential ginsenosides including Rg2, Rb1, Rd and Re, however, which were present in a relatively high amount in AGs from Canada. It was difficult to directly rank and compare the quality of AGs from different cultivation regions simultaneously based on these differential ginsenosides. Therefore, an unsupervised principal component analysis (PCA) on the contents of nine ginsenosides was further conducted to reveal the overall quality difference and relationship of AGs from different cultivation regions (Fig. 1). Our PCA result turned out that AGs from Shaanxi and US were clustered closely and separated from other five cultivation regions. AGs grown in Heilongjiang, Jilin and Shandong had similar ginsenoside profiling in terms of nine analytes. The quality of AGs grown in Beijing was similar to that from Canada. Our classification on different origins of cultivated AGs was almost consistent with those of Huang *et al*.^25^ and Wang *et al*.^30^. Heatmap analysis of Huang *et al*.^25^ demonstrated that domestic AGs formed two chemoecotypes: inside Shanhaiguan group containing Beijing and Shandong provinces and outside Shanhaiguan group including Jilin, Heilongjiang and Liaoning provinces. PCA result of Wang *et al*.^30^ showed that ginsenosides in the roots of *P. quinquefolius* in Beijing, Jilin, and Heilongjiang regions were more similar than those from United States, Shandong and Shaanxi.

### Effects of AG decoctions from different cultivation regions on splenic antioxidant levels in irradiated mice

**Figure 2:**
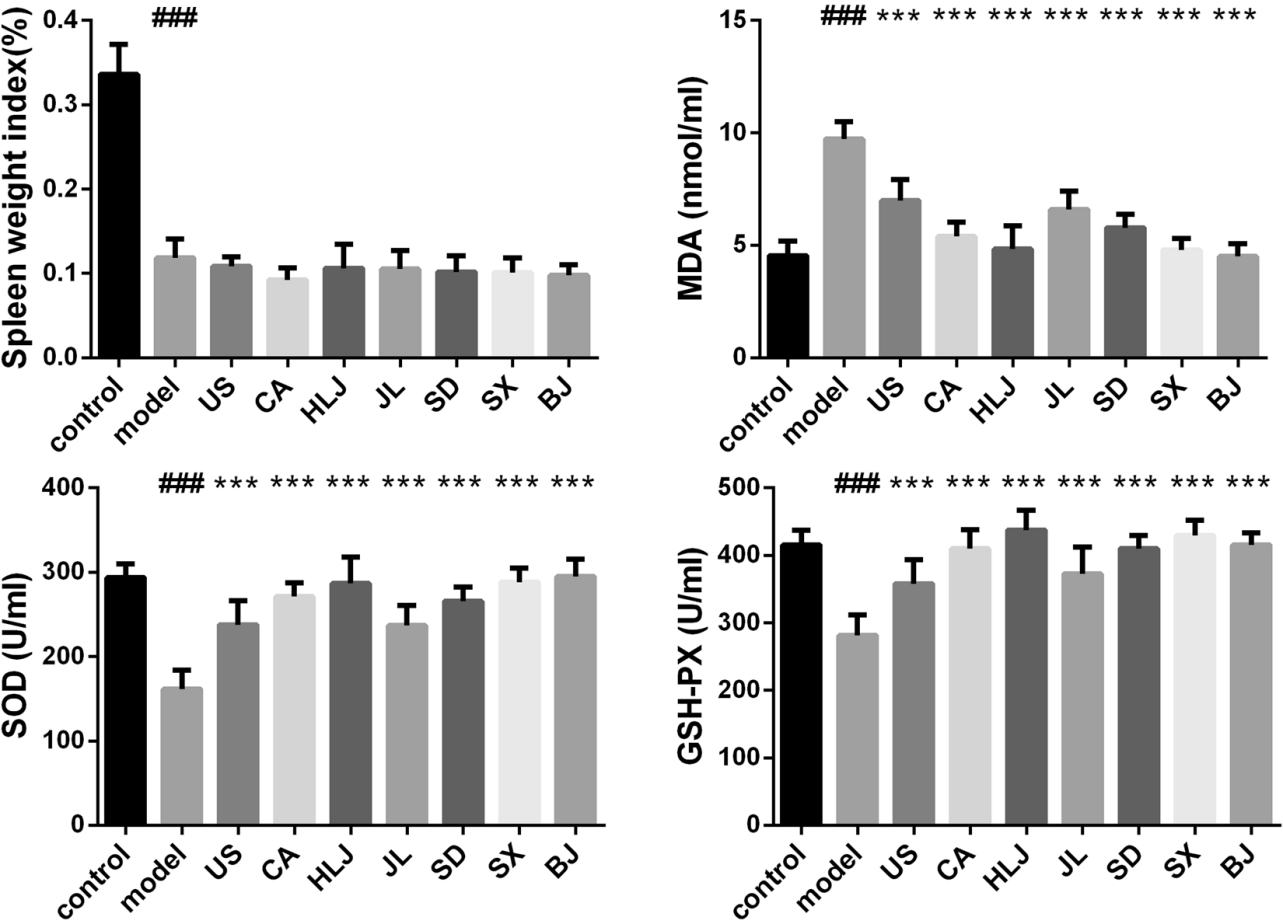
Effects of AG decoctions on spleen weight index, MDA level and antioxidant enzymatic activities in irradiated mice. The names on x-axis here and in Figs 3–8 were denoted as: control (C), normal control group without irradiation and AG administration; model (M), irradiated model mice without AG administration; US, CA, HLJ, JL, SD, SX, and BJ: abbreviated name of the cultivation place of AG decoction given to irradiated mice. ^###^*p* < 0.001 meant significant difference between model and control groups; ***p < 0.001 meant significant difference between model group and AG-pretreated radiated groups.

Superoxide dismutase and glutathione peroxidase (GSH-Px) are two important enzymatic members in antioxidant defense system against the formation of reactive oxygen species (ROS) and free radicals in living beings. Malonyldialdehyde (MDA) is produced during the peroxidation of polyunsaturated fatty acids and used as an evaluation marker of various oxidative stresses. Ionizing radiation led to the significant alterations of living beings in activities of antioxidant defense enzymes such as SOD and GSH-PX and MDA level. It was observed that administration of American Ginseng Capsule (AGC) suspension could significantly decrease liver MDA level while increase the contents of liver SOD and GSH in rats exposed to 900 MHz cell phone electromagnetic radiation^48^. Preadministration of AG decoction for 14 days also showed the similar changing trends of MDA and SOD content in mouse lungs subjected to one- hour long 20 Gy of x-ray irradiation^49^. Spleen is one of the important and radiosensitive immune system organs. Therefore, we investigated and compared MDA level and SOD and GSH-PX activities in spleen of irradiated model mice and AG-treated irradiated mice. Similar results of AG’s protection on mouse spleen antioxidant defense system from γ-ray injury were observed as in electromagnet irradiated mouse liver^48^ and X-ray irradiated mouse lung^49^. Compared with the control group, five day’s consecutive total body irradiation (TBI) significantly increased MDA content and decreased the spleen weight index (spleen weight/body weight, SWI) and the SOD and GSH-PX activities in irradiated model mice (Fig. 2). This indicated that oxidative stress and damage to mouse spleen was induced by the total of 5 Gys sublethal irradiation dose. Except that spleen weight index, the irradiation-caused changes of MDA, SOD and GSH-PX levels could be reversed in various degrees by preadministration of AG decoctions. However, AGs from different cultivation regions displayed the differential antioxidant capacities on radiation. Except that AGs from USA, Jilin and Shandong provinces, AGs from Canada, Heilongjiang, Shaanxi and Beijing provinces of China could restore the levels of splenic MDA and SOD in irradiated mice to the status of the normal control. Splenic GSH-PX in irradiated mice could be increased greatly by all the seven AG decoctions. However, it could not be completely restored in US and JL group. GSH-PX levels in these two groups were slightly lower than in the control.

### Effects of AG decoctions from different cultivation regions on cytokine levels in irradiated mice

**Figure 3:**
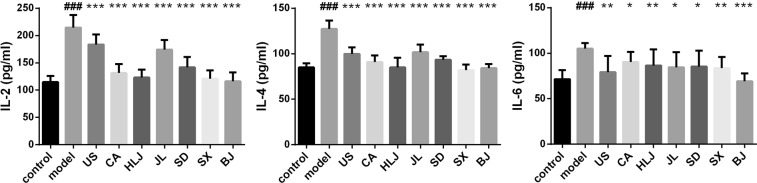
Effects of AG decoctions on splenic cytokine levels in irradiated mice. ^###^*p* < 0.001 meant significant difference between model and control groups; *p < 0.05, **p < 0.01, ***p < 0.001 meant significant difference between model group and AG-pretreated radiated groups.

**Figure 4:**
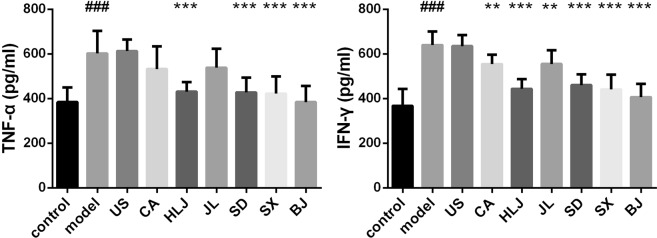
Effects of AG decoctions on serum cytokine levels in irradiated mice. ^###^*p* < 0.001 meant significant difference between model and control groups; **p < 0.01, ***p < 0.001 meant significant difference between model group and AG-pretreated radiated groups.

Radiation exposure could induce immune and inflammatory responses of irradiated tissues/organisms^7^. Cytokines play important roles in radiation-induced immune and inflammatory responses and are modulated by ionizing radiation. Proinflammatory cytokines interleukin-6 (IL-6) and tumor necrosis factor-α (TNF-α) were elevated by irradiation and regarded as the early responsive biomarkers of radiation injuries^50,51^. They were also observed in our study to be significantly enhanced, respectively in the spleen and serum of irradiated mice, compared to the unirradiated control group (Figs 3 and 4), which indicated that the 5 Gys of TBI caused inflammation responses in irradiated mice. Th1-type IL-2 and Th2-type IL-4 were also obviously increased in splenocytes of irradiated model mice. Th1-type cytokine interferon-γ (IFN-γ) was associated with the pathogenesis of chronic inflammatory and autoimmune diseases and found positively associated with the increased probabilities of radiation-related acute hematologic/organ toxicities^52^. IFN-γ was elevated by about 1.7 folds in sera of irradiated mice than the control, which indicated that our radiation treatment induced the possible hematologic toxicity.

Like its antioxidant capacity, the modulating capacities of AGs on cytokine levels in irradiated mice still showed the cultivation origin-related difference (Figs 3 and 4). Among these five analyzed cytokines, pretreatment of AGs from all the seven cultivation regions had the potentials to significantly prevent the enhancement of splenic IL-2, -4 and -6 in irradiated mice (Fig. 3), of which IL-6 was found to be greatly inhibited in X-ray radiated mouse lung by pretretment of *Panax ginseng* extract^40^. Compared to the control group, the levels of splenic IL-2 in CA, HLJ, SX and BJ groups showed no difference from the control and were restored to the level of the control mice. The irradiated mice with pretreatment of US, JL and SD-AGs had the higher IL-2 levels than the control. IL-4 level was significantly higher only in US and JL groups compared to the normal control group. Levels of IL-6 did not differ significantly between the normal control and AG-pretreated radiation groups except CA group. Compared to the model group, the levels of serum TNF-α in irradiated mice receiving AG decoctions from Heilongjiang, Shandong, Shaanxi and Beijing were significantly declined and restored to the normal level. However, there was no significant difference in serum TNF-α between US, CA and JL groups and the model group, indicating that the increasing trend of serum TNF-α in irradiated mouse could not be stopped by pre-administration of AG decoctions from USA, Canada and Jilin. Compared to the model group, the other serum cytokine IFN-γ was observed to significantly decline in all AG-treated irradiated groups except US group. However, its levels in sera of these AG-treated groups including CA, HLJ, JL, SD and SX were still significantly higher than the value in the control group. There was no significant difference in serum IFN-γ between BJ group and the control, indicating that Beijing-originated AG could have the potential to remarkably reduce radiation toxicities on hematologic or other organs, however, which need to be further histological investigations.

### Effects of AG decoctions from different cultivation regions on hormone levels in irradiated mice

**Figure 5:**
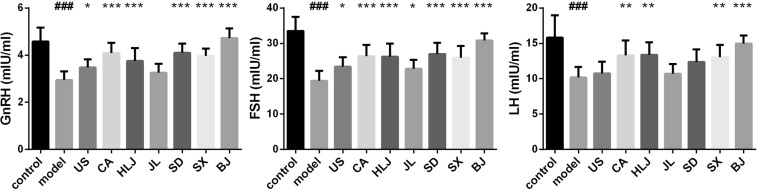
Effects of AG decoctions on brain hormone levels in irradiated mice. ^###^*p* < 0.001 meant significant difference between model and control groups; *p < 0.05, **p < 0.01, ***p < 0.001 meant significant difference between model group and AG-pretreated radiated groups.

**Figure 6:**
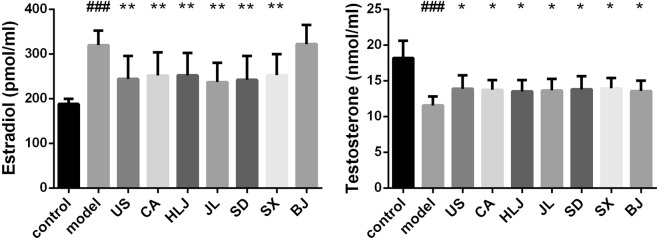
Effects of AG decoctions on testis steroid hormone levels in irradiated mice. ^###^*p* < 0.001 meant significant difference between model and control groups; *p < 0.05, **p < 0.01 meant significant difference between model group and AG-pretreated radiated groups.

Gonadotrophin-releasing hormone (GnRH), follicle stimulating hormone (FSH) and luteinizing hormone (LH) play important roles in development, growth, pubertal maturation, gonadal maturation and fertility of the body. GnRH is synthesized and released from GnRH neurons within the hypothalamus. Gonadotropins FSH and LH are synthesized and released from the anterior pituitary, which are modulated by neuropeptides such as GnRH. Radiation induces hypothalamic–pituitary axis (HPA) dysfunction especially in the treatment of nasopharyngeal carcinoma and intracranial neoplasms^53,54^. American ginseng could reduce serum FSH and LH concentrations while promoted E2 secretion in ovarian aged or premature mice^55^, which indicated that AG has the capability to protect HPA function via regulation of hormone levels. Compared with the control group, our work showed that three brain homones together with testicular testosterone were all markedly declined in irradiated model mice by total body irradiation, whereas testicular estradiol was raised (Figs 5 and 6). Generally, pre-administration of AG decoction could reverse irradiation-induced changes of these hormones, although some cultivation regions of AGs showed no or only partial effectiveness on some hormones. For example, pre-administration of Beijing-originated AG decoction could restore the levels of brain GnRH, FSH and LH in irradiated mice to the levels of the control mice and could not inhibit the radiation-induced decrease of testicular estradiol. All the seven AG-administrated groups showed the significant increase in brain FSH and testicular testosterone, although both of which were generally lower than the control group.

### Principal component analysis and hierarchical clustering analysis of the biochemical parameters

**Figure 7:**
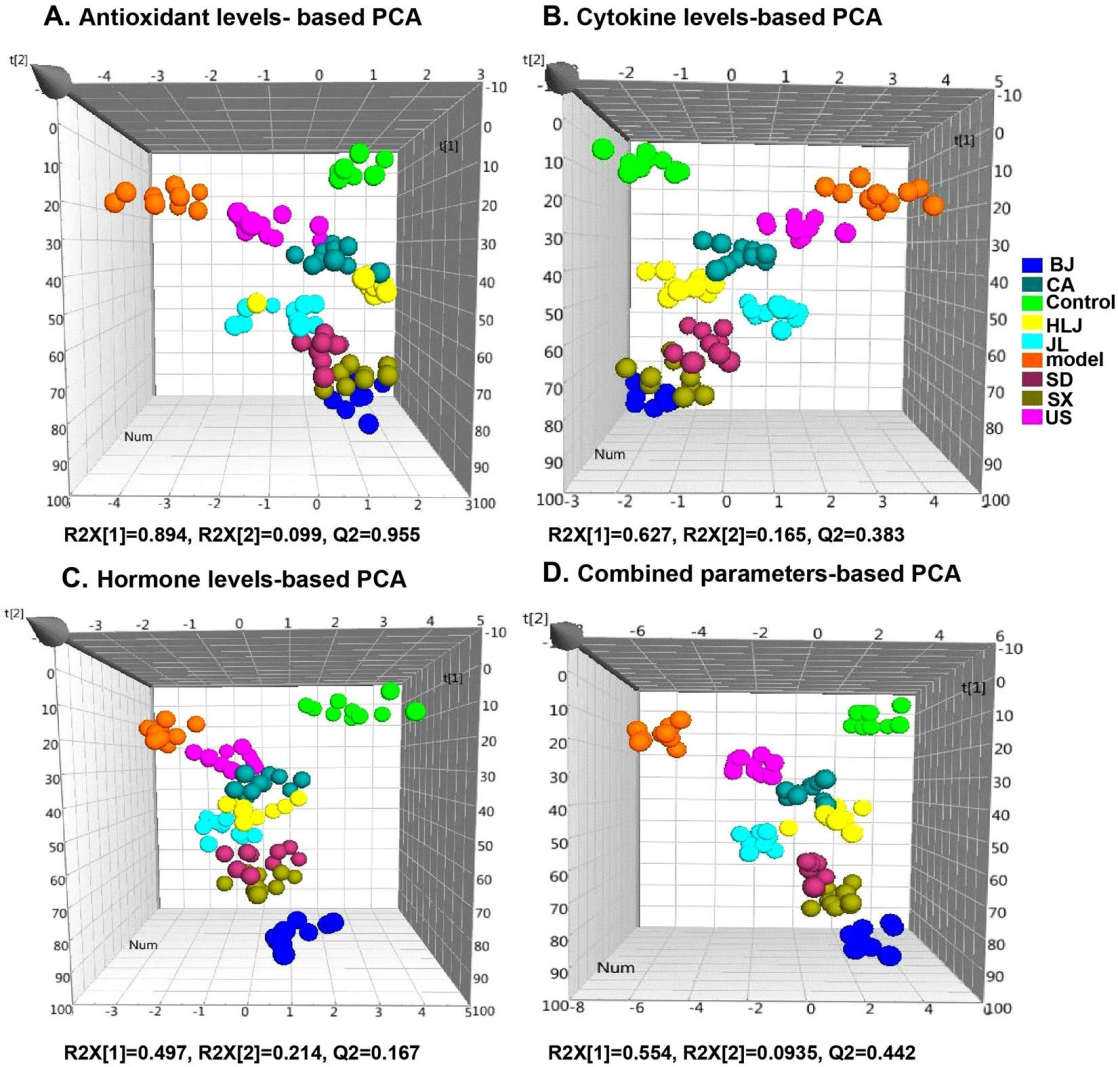
PCA score plots of mouse biochemical measurements showed the differential pharmacological effects of AGs from different cultivation regions.

**Figure 8:**
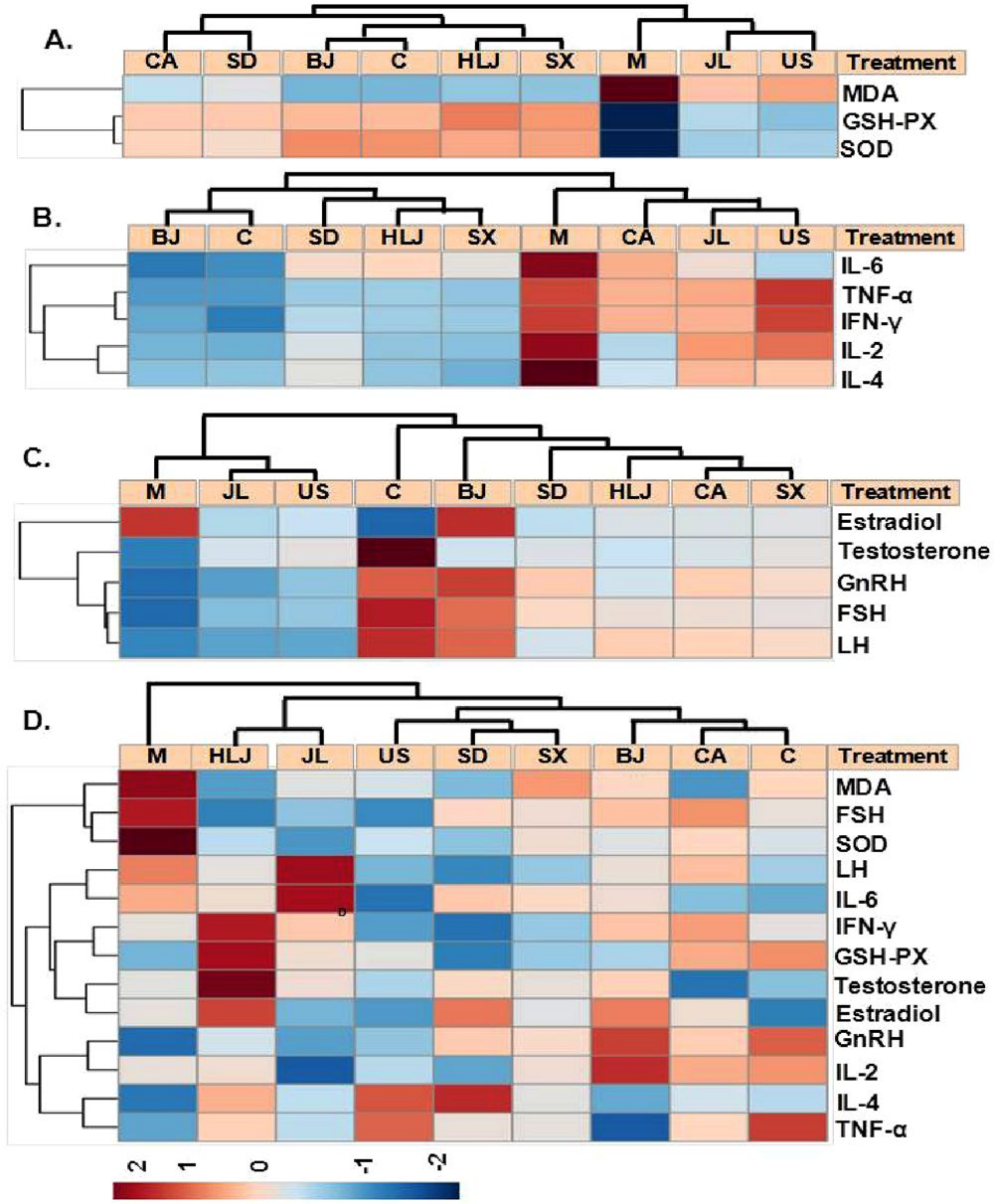
Heatmaps of mouse biochemical measurements showed the differential pharmacological effects of AGs from different cultivation regions. (**A**) Antioxidant levels-based heatmap; (**B**) cytokine levels-based heatmap; (**C**) hormone levels-based heatmap; (**D**) combined 13 parameters-based heatmap. The relative expression of analyzed biochemical measurements varied from −2.0 to 2.0.

Although our above comparisons of individual biochemical parameters revealed the relative radioprotective effects of AGs from different cultivation regions, however, it is hard and impossible to make a clear and consistent conclusion just based on some or few of the obtained measurements, for example, testicular estradiol and brain hormones. Therefore, to obtain a better overall pharmacological effect difference of AGs from different cultivation regions, we further performed principal component analysis and hierarchical clustering analysis on these data (Figs 7 and 8). PCA results (Fig. 7) showed that there were good separations among the non-irradiated normal control group, the model group and AG groups and even within AG groups. The antioxidant levels-based PCA (Fig. 7A) resulted in two principal components with R2X [1] = 0.894, R2X [2] = 0.099 and Q2 (cum) = 0.955. Cytokine levels-based and hormone levels-based PCAs (Fig. 7B,C) also generated two principal components, revealing about 62.7% and 49.7% of the total variance, respectively. The further heatmaps which were constructed separately by these three types of biochemical parameters (Fig. 8A–C) revealed the similar relationships of different cultivation regions of AGs. Figure 8A,C showed that nine treatments were clustered into two major groups: US, JL and model group and the second cladom containing BJ, control, HLJ, SX, SD and CA group. BJ group was clustered with the control, indicating Beijing-cultivated AGs had the best antioxidant and hormone-adjusting capacities against radiation. AGs cultivated in Canada, Jilin and USA had the poor immunomodulatory capacity in response to radiation and they were clustered with the model group (Fig. 8C). While these three types of biochemical measurements were combined, PCA and heatmap results (Figs 7D and 8D) showed that the model group was separated from both the control and AG-administrated irradiated groups, indicating that American ginseng had the radioprotection effects due to its antioxidant and immunomodulatory properties and the modulation of hypothalamic–pituitary–gonadal (HPG) system^56,57^. However, the radioprotection effects varied among different cultivation regions of AGs. Generally, Beijing and Canada-cultivated AGs had the best radioprotection in irradiated mice. Heilongjiang and Jilin-originated AGs showed the similar pharmacological effects whereras USA, Shandong and Shaanxi-grown AGs had the closer pharmacological effects. This biochemical parameters-based classification of AGs from seven cultivation regions was nearly consistent with serum metabolome-based analysis^58^, even though it demonstrated that AG had no effect in modulation of the radiation-altered metabolites in serum.

### Correlation analysis of ginsenoside contents and biochemical measurements

In this study, the ginsenoside content-based quality evaluation of AGs from the different cultivation regions was similar as the radioprotective effects-based findings. Ginsenosides such as Rb1, Rg1, Rg2, Rg3, Re and Rd were demonstrated to have the anti-oxidant, anti-inflammatory, immunopotentiating and neuroprotective effects^13^. *In-vitro* and *in-vivo Panax ginseng* experiments of individual ginsenosides showed that ginsenosides Rg1, Rg2, Rg3, Rb1, Rb2, Rb3, Rc, Rd and Re had the radioprotection against radiation-induced ROS, oxidative stress, aging, apoptosis and other side effects^36,38,39,59–66^. Pure ginsenoside experiments revealed that Rg1^60^, Rb2^62^, Rb3^64^ and Rg2^65^ significantly attenuated irradiation-induced changes of SOD, MDA and GSH, which indicated that the varying content of some ginsenosides in AG extracts was possibly correlated with the varying values of the assayed biochemical parameters among AG treatments. Therefore, we further performed the pearson correlation analysis of AG ginsenoside contents and mice biochemical measurements to evaluate the contributions of AG bioactive compounds in AG extract on AG radioprotection effects (Table 2). The result showed that only Rb3 and Rd were significantly correlated with antioxidant activities and cytokine levels. Rb3 and Rd negatively correlated with GSH-PX (r = −0.865) and SOD (r = −0.786), respectively. They both showed the high positive correlations with serum cytokines TNF-α and IFN-γ. Although they were highly correlated with IL-2 and IL-4, however, the significant correlations were only found between Rb3 and IL-2 and Rd and IL-4. The poor association for most analyzed ginsenosides and mice biochemical measurements might be attributed to the significant but tiny difference of ginsenoside contents among some cultivation regions. A second reason is that most mice biochemical measurements except TNF-α and IFN-γ did not change greatly among different AG treatments and show no proportion to their respective contents in term of the individual ginsenosides in the AG extract. In addition, the pharmacological effects of a herbal medicine reflect the integrative effects of many constituents with synergistic or antagonistic effects within itself. Lee *et al*.^36^ found that the same amount of individual ginsenosides were supplied separately and had the varying effects on the same studied pharmacological traits. Therefore, the effects of the ginsenoside with the lower content in a cultivation region would possibly be enhanced or weakened by another varying amount of ginsenosides or other unquantitated radioprotectors such as polysaccharides which also had antioxidant and immunoregulatory activities^67,68^. In this sense, it was reasonable to observe the low correlation between the content of a ginsenoside in AG extract and the biochemical data in our work. In terms of the overall effect, Rc, Rd and Re had a major radioprotective effect in irradiated mice, based on the same dose experiment of pure ginsenosides^36^. However, in order to unveil the relative radioprotective effects of AG ginsenosides in AG extract, individual ginsenosides should be separated from AG roots and simultaneously studied in the future with the same dose or their real proportion in AG extract.

**Table 2:** Correlation coefficients of AG ginsenoside content and the biochemical measurements in AG-administered mouse.

	Rg1	Rg2	Rg3	Rb1	Rb2	Rb3	Rc	Rd	Re	Rg1 + Rb1 + Rd	Total content of nine ginsenosides
GSH-PX	0.057	−0.163	−0.129	−0.112	−0.365	−0.865*	−0.419	−0.679	−0.364	−0.199	−0.264
SOD	−0.012	−0.118	−0.135	−0.150	−0.488	−0.744	−0.149	−0.786*	−0.296	0.160	−0.272
MDA	−0.028	0.081	0.236	0.117	0.409	0.723	0.233	0.714	0.243	−0.203	0.229
GnRH	−0.284	0.098	−0.294	0.045	−0.651	−0.492	0.260	−0.607	−0.021	0.006	−0.073
FSH	−0.338	0.242	−0.316	0.035	−0.509	−0.514	0.099	−0.500	−0.079	−0.0024	−0.098
LH	−0.030	0.038	−0.218	0.079	−0.438	−0.556	−0.010	−0.500	−0.034	0.037	−0.036
Testosterone	0.015	−0.668	0.254	−0.426	−0.597	−0.026	0.552	−0.250	−0.243	−0.361	−0.358
Estradiol	−0.277	0.345	−0.309	−0.039	−0.268	−0.112	0.228	−0.714	0.048	−0.023	−0.064
IL-2	−0.040	0.132	0.220	0.050	0.477	0.767*	0.203	0.714	0.211	0.105	0.180
IL-4	−0.073	0.266	0.041	0.265	0.513	0.750	0.131	0.821*	0.375	0.301	0.363
IL-6	0.525	−0.438	0.189	0.352	0.156	−0.137	−0.337	0.429	0.166	0.314	0.316
TNF-α	0.433	−0.225	0.463	0.355	0.392	0.851*	0.414	0.857*	0.537	0.444	0.508
IFN-γ	0.393	−0.220	0.453	0.359	0.348	0.838*	0.447	0.779*	0.535	0.443	0.504

Note: *indicated significant correlation (*p* < 0.05) between the ginsenoside content and the assayed mouse biochemical level.

## Conclusions

*In-vitro* and *in-vivo* experiments showed that American ginseng could attenuate radiation-induced DNA damage and liver injuries. Our results revealed that pre-administration of AG decoctions could restore the irradiation-induced changes of antioxidant enzymatic activity, cytokine level and hormone level in irradiated mice, which suggested that AG’s radioprotective effects were attributed to its antioxidative, immunomodulatory and anti-inflammatory properties. However, this radioprotection varied among AGs of different cultivation regions. The biochemical measurements-based PCA and heatmap clustering of AGs from seven cultivation regions showed that Beijing and Canada-cultivated AGs were grouped with the control mice, indicating that they possessed the best radioprotection in this study. Heilongjiang and Jilin-originated AGs exhibited the similar pharmacological effects while USA, Shandong and Shaanxi-grown AGs had closer pharmacological effects. Although the low pearson correlation was found between ginsenoside contents in AG extracts of different cultivation regions and their corresponding biochemical measurements in irradiated mice, the overall pharmacological (radioprotective) effect difference presented among AGs of seven cultivation regions was nearly similar to ginsenoside content- and serum metabolome-based results, which provided the scientific basis that we could evaluate the pharmacological difference of AG samples based on measurements of some important multiple bioactive compounds in plants. AG polysaccharides are another important compounds with antioxidant and immunoregulatory activities and scarcely studied among different AG samples. Thus, it would be interesting to study its quality difference among different origins of AGs and its contribution to pharmacological difference of different origins of AGs.

## Materials and Methods

### Plant material

Thirty-eight four-year-old main root samples of American ginseng (AG) were collected from American, Canada and five provinces of China including Heilongjiang, Jilin, Shandong, Shaanxi and Beijing (Table 3). The domestic AG roots were collected in October, 2015. The fresh taproots of American ginseng were dried at 30 °C, then ground and passed through a 60 mesh size. Root powder was kept at 4 °C till extraction.

**Table 3:** Cultivation region of *P. quinquefolium* roots used in this study.

No.	Cultivation region	No.	Cultivation region
US01	Wisconsin, USA	JL02	Baishan, Jilin, China
US02	Wisconsin, USA	JL03	Baishan, Jilin, China
US03	Wisconsin, USA	JL04	Baishan, Jilin, China
US04	Wisconsin, USA	JL05	Baishan, Jilin, China
US05	Wisconsin, USA	JL06	Baishan, Jilin, China
US06	Wisconsin, USA	JL07	Yanbian, Jilin, China
US07	Wisconsin, USA	JL08	Tonghua, jilin, China
US08	Wisconsin, USA	SD01	Weihai, Shandong, China
CA01	Quebec, Canada	SD02	Weihai, Shandong, China
CA02	Quebec, Canada	SD03	Weihai, Shandong, China
CA03	Quebec, Canada	SD04	Weihai, Shandong, China
CA04	Quebec, Canada	SD05	Weihai, Shandong, China
CA05	Quebec, Canada	SX01	Hanzhong, Shaanxi, China
HLJ01	Jixi, Heilongjiang, China	SX02	Hanzhong, Shaanxi, China
HLJ02	Jixi, Heilongjiang, China	SX03	Hanzhong, Shaanxi, China
HLJ03	Suihua, Heilongjiang, China	SX04	Hanzhong, Shaanxi, China
HLJ04	Harbin, Heilongjiang, China	SX05	Hanzhong, Shaanxi, China
HLJ05	Shuangyashan, Heilongjiang, China	BJ01	Huairou District, Beijing, China
JL01	Baishan, Jilin, China	BJ02	Huairou District, Beijing, China

The samples were named after their originating country or province of China. The samples US01-US07 and CA01-CA04 were purchased from Beijing Tongrentang Co., Ltd, representing different batches from American and Canada, separately. US08 and CA05 were collected directly from American and Canada. The series number followed the abbreviated province of China meant the AG roots from different farms of the same city or different cities of the same province.

### UPLC-UV determination of ginsenoside contents in AG roots

HPLC-grade acetonitrile (ACN) and methanol were purchased from Thermo Fisher (USA) and Honeywell Co.Ltd., separately. Standards of nine ginsenosides including Rb1 (GR-16021907), Rb2 (GR-16032211), Rb3 (GR-16032211), Rc (GR-15111902), Rd (GR-16012503), Re (GR-16012701), Rg1 (GR-16022407), Rg2 (GR-16011604) and Rg3 (GR-16030711) were purchased from Chengdu Must Biotechnology Co. Ltd., China (http://cdmust.guidechem.com/). The purities of all the standards were higher than 98%. The purified water from Wahaha was used throughout the experiment. Other reagents of analytical grade were obtained from Beijing Chemical Industry Inc., China.

The concentrations of ginsenosides Rb1, Rb2, Rb3, Rc, Rd, Re, Rg1, Rg2 and Rg3 in the mixed working stock standard solution (labeled as s1) were, separately, 1.756 mg/ml, 0.029 mg/ml, 0.024 mg/ml, 0.16 mg/ml, 0.178 mg/ml, 1.36 mg/ml, 0.206 mg/ml, 0.0117 mg/ml and 0.02 mg/ml in 5 mL of methanol. To make calibration curves, seven series concentrations were then separately prepared by diluting the working stock solution s1 with 0.7 fold difference. That is, 3.5 mL of former standard solution and 1.5 mL of methanol were taken to make 5 mL of standard solution.

0.5 gram of each root powder sample was accurately weighed and ultra-sonicated once at the 100 Hz with 10 mL of chromatographic grade methanol at room temperature for 30 min. The extract was made up to the same volume with methanol and then centrifuged at 10000 rpm for 10 min at 4 °C. The supernatant was filtered with a 0.22 μm millipore membrane filter. One mL of the filtrate was transferred into a sample vial for the below UPLC-UV determination of the above nine ginsenosides. Three replicates of each location of root sample were prepared.

Two microliters of ginsenoside standards and filtered AG root extracts were run at 35 °C on a Waters ACQUITY ultra performance liquid chromatography (UPLC, Ireland) system coupled with a Waters BEH C18 column (1.7 µm, 2.1 × 100 mm). The mobile phase consisted of acetonitrile (A) and H2O (B). The optimized gradient conditions were set as: 0–3 min, 19% A; 3–5 min, 19–21% A; 5–10 min, 21–24% A; 10–12 min, 24–29.3% A; 12–14 min, 29.3% A; 14–16 min, 29.3–32% A; 16–18 min, 32%A; 18–20 min, 32–43% A; 20–23 min, 43–60% A; 23–24 min, 60% A; 24-25 min, 60-19% A. The flow rate was 0.4 mL/min for the first five minutes and then 0.3 mL/min for the rest time. The UV wavelength of the detector was set at 203 nm. Each extract was run twice. The contents of these nine ginsenosides in AG root samples were calculated using their respective standard curves (Table 4). Calibration curve of each standard was constructed by plotting the logarithm of its UPLC-UV peak area versus the logarithm concentration since peak area and concentration displayed the linear relationship after log transformation. Ginsenoside content in AG root was expressed as the weight of the assayed ginsenoside relative to the root dry weight (mg/g).

**Table 4:** Linear regression equations, correlation coefficients and linear ranges of nine ginsenosides.

Ginsenoside	Linear regression equation	Correlation coefficient (r^2^)	Linear range (mg/mL)
Rb1	y = 0.9327x − 5.7217	0.9996	0.207∼1.756
Rb2	y = 1.0128x − 6.1414	0.9991	0.003∼0.029
Rb3	y = 1.0125x − 6.257	0.9990	0.003∼0.024
Rg1	y = 0.9163x − 5.6615	0.9994	0.024∼0.206
Rg2	y = 1.0207x − 6.4379	0.9996	0.001∼0.012
Rg3	y = 0.9788x − 6.2113	0.9990	0.002∼0.020
Rc	y = 0.9313x − 5.8274	0.9991	0.019∼0.160
Rd	y = 0.9358x − 5.9287	0.9996	0.021∼0.178
Re	y = 0.9245x − 5.6108	0.9995	0.160∼1.360

### Preparation of AG decoctions for mice administration

To investigate the pharmacological difference and thus evaluate the quality difference of AGs from five provinces of China, USA and Canada, the pulverized root samples from different places of the same province of China or from different batches of USA and Canada were mixed equally into seven AG samples representing AG products from USA, Canada, Heilongjiang (HLJ), Jilin (JL), Shandong (SD), Shaanxi (SX) and Beijing (BJ) provinces of China. Then, 20 grams of the well-mixed root powder was weighed and extracted for three times. Each time, the root powder or residue was soaked in 500 mL of distilled water for one hour and boiled for 45 min. The pooled filtrate from three extractions was concentrated to 100 mL and kept at 4 °C till the commencement of the below mice administration.

### Administration of AG decoction and ^60^Co γ-irradiation

The experiments including AG administration and irradiating mice were performed in accordance with Qin’s^53^. 108 ICR male mice weighing 20 ± 2 g were obtained from Peking University Health Science Center, Beijing, China (No. SCXK (Jing) 2014-0006) and housed at the animal center of IMPLAD, Beijing, China. The housing conditions were set as: 22–23 °C, 55 ± 5% humidity and 12-h light/dark cycle. The mice were acclimatized for one week and then randomly divided into normal control (NC) group, model control (MC) group and seven AG-administered groups. The seven administered mice groups were separately injected with seven cultivation regions of AG decoctions for 28 days prior to irradiation. The daily intragastrical injection dose for each experimental mouse was 0.2 mL AG decoction per 10 g of body weight. NC and MC mice received the same amount of distilled water daily as AG-administered groups. The total injection volume of the individual mouse was weekly adjusted based on its weekly body weight. After 28 days’ chronic administration, MC and AG-administered groups were daily whole body irradiated with 1.0 Gy ^60^Cobalt γ-rays for five consecutive days. The Guidelines of National Health Institutes of China for the Care and Use of Laboratory Animals (Certificate No. SYXK2013-0023 (Jing)) was obeyed throughout the animal experiment. The protocols were approved by our institutional ethics review board (Institute of Medicinal Plant Development, Chinese Academy of Medical Sciences).

### Biochemical analyses

An enzyme-linked immunosorbent assay (ELISA) method was employed to measure the levels of the following antioxidant, cytokine and hormone parameters.

On the next day of the last ^60^Cobalt radiation, all the mice were sacrificed by cervical dislocation. Blood was collected through eye vessels and centrifuged at 4 °C at 3500 rpm for 10 min to obtain serum supernatant. Sera were then kept at −80 °C until the measurement of cytokines: interferon-γ (IFN-γ) and tumor necrosis factor-α (TNF-α).

The activities of glutathione peroxidase (GSH-Px) and superoxide dismutase (SOD) and the concentrations of malondialdehyde (MDA) and interleukins (IL-2, -4 and -6) were assayed in the spleen. The levels of gonadotrophin-releasing hormone (GnRH), follicle stimulating hormone (FSH) and luteinizing hormone (LH) were measured in the brain. The sex steroid hormones including Testosterone (T) and Estradiol (E2) were quantified in the testis. On the sampling day, spleen, brain and testis were separately collected, rinsed with physiological saline, dried on tissue paper and immediately stored at −80 °C until use. The organ tissues were thawed and homogenized in the ice-cold physiological saline to make 10% (g/ml) homogenates. The homogenate was centrifuged at 3,000 rpm at  4 °C for 10 min and then the supernatant was collected for the quantitation of total protein and ELISA assays. The protein concentrations of homogenate supernatants were determined using BCA method and subsequently normalized to the equal content before ELISA analysis.

Except that the mouse ELISA kits for antioxidant activities were obtained from NanJing JianCheng Bioengineering Institute, China (http://www.njjcbio.com/), all other ELISA kits were purchased from Beijing Donggeboye company (http://www.dg-reagent.com/). The procedures for all measurements were conducted according to the protocols of the corresponding ELISA kits.

### Statistical analysis

All data were represented as mean ± standard deviation (SD). The significance analysis was assessed with one-way ANOVA in excel or SPSS software. P < 0.05 was considered significant between the pairwised comparison. To reveal the relationships of AGs from different cultivation regions, principal component analyses based on AG ginsenoside contents and mouse biochemical measurements were conducted in SIMCA-P software (V.13.0, Umetric, Umea, Sweden). The unit variance scaling was applied to process the data before PCA. Meantime, the mouse physiological data were subjected to the heatmap analysis, which was implemented in MetaboAnalyst (http://www.metaboanalyst.ca/). Pearson correlation analyses of AG ginsenosides and AG pharmacological effects were conducted in SPSS (version 22.0) for accessing the influence of AG bioactive compounds on AG pharmacological effects.

## Supplementary information

Supplementary dataset 1 2

## Data Availability

All the raw data could be provided if required.
