# Supplementary material for: Quality evaluation of Panax quinquefolium from different cultivation regions based on their ginsenoside content and radioprotective effects on irradiated mice
Source: Sci Rep. 2019 Jan 31;9:1079. doi: 10.1038/s41598-018-37959-9 (PMC6355895; doi:10.1038/s41598-018-37959-9)

Quality evaluation of *Panax quinquefolium* from different cultivation regions based on their ginsenoside content and radioprotective effects on irradiated mice

Dengqun Liao[1#], Chan Jia[1#], Peng Sun[1], Jianjun Qi[1], Xian'en Li[1*]

[1]Institute of Medicinal Plant Development, Chinese Academy of Medical Sciences & Peking Union Medical College, Beijing 100193, PR China
*Corresponding author:
Xian'en Li
Tel.: +86-10-57833429
Email: xianenli@yeah.net

[#]These authors contributed equally to this work.

Supplementary Table 1 Optimization of UPLC-UV conditions for simultaneous determination of nine AG ginsenosides.

Chromatographic factor	Compared parameter	Opitimized conditions	Main advantages or reasons selected
Column	ACQUITY UPLC ® BEH C18 (2.1*100mm, 1.7μm), ACQUITY UPLC ® BEH C18 (2.1*50mm, 1.7μm)	2.1*100mm, 1.7 μm	Close speed but better separation
λ_{UV}	203 nm	203 nm	Used by many literatures and Chinese Pharmacopoeia
Mobile phase	Methanol-H_2O, ACN-H_2O; ACN- 0.1% FA in H2O	ACN-H_2O	RT, lower column pressure and damage
	linear gradient*	See in the method	Modified based on Chinese Pharmacopoeia and Xie
Running time*	20, 25, 30 min	See in the method	
Flow rate*	0.2, 0.3and 0.4 mL/min	See in the method	*Shorter elution time, Best separation, less damage to column

- The optimization of whole running time, running time of each gradient region and gradients were mainly conducted at 0.3 mL/min. The final adopted conditions were determined by cross-checking these three factors (*) on separation of ginsenoside standards especially on Rg1 and Re.

Xie, C.X., Suo, F.M., Jia, G.L., Song, J.Y., Huang, L.F. & Chen, S.L. Correlation between ecological factors and ginsenosides. *Acta Ecologica Sinica* **31(24)**, 7551-7563.

Supplementary Figure 1 UPLC-UV chromatographs of blank (a) , standard mixture (b) and AG extract (c) under the optimized chromatographic conditions.

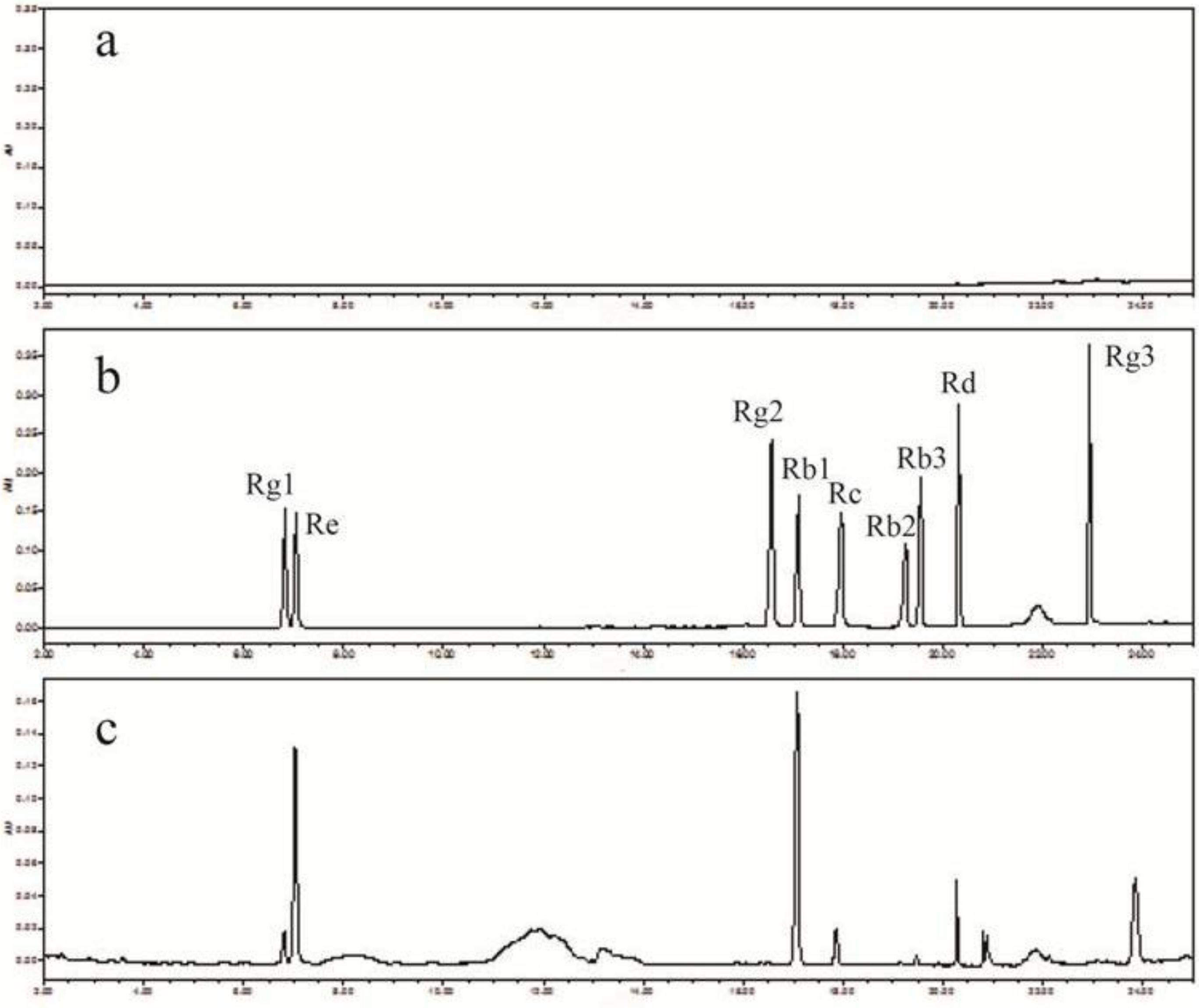

Supplement: Supplementary file 1 — Supplementary dataset 1 2 [file 41598_2018_37959_MOESM1_ESM.pdf]
